# Diet quality and its associated factors among adolescents in high schools of Rawalpindi and Islamabad

**DOI:** 10.3389/fpubh.2025.1651903

**Published:** 2025-11-10

**Authors:** Fatima Falak, Abdul Momin Rizwan Ahmad, Humaira Mahmood, Zoha Imtiaz Malik, Syed Hassan Bin Usman Shah, Juweria Abid, Maryam Irfan

**Affiliations:** 1 Department of Public Health, Armed Forces Postgraduate Medical Institute (AFPGMI), National University of Medical Sciences (NUMS), Rawalpindi, Pakistan; 2 Department of Human Nutrition and Dietetics, NUST School of Health Sciences, National University of Sciences & Technology (NUST), Sector H-12, Islamabad, Pakistan; 3 Department of Health Sciences, University of York, York, United Kingdom; 4 Department of Public Health, Health Services Academy, Islamabad, Pakistan; 5 The Kirby Institute, University of New South Wales, Sydney, NSW, Australia; 6 Department of Nutrition and Dietetics, National University of Medical Sciences (NUMS), Rawalpindi, Pakistan

**Keywords:** adolescents, diet quality, diet quality questionnaire, socioeconomic status, body image, academic performance, high school students

## Abstract

**Introduction:**

Adolescence is a critical developmental stage during which dietary behaviors have profound impacts on physical health, mental well-being, and academic performance.

**Objective:**

The objective of this study was to examine the associations between diet quality and academic performance, body image perceptions, and demographic factors.

**Methods:**

*n* = 281 adolescents aged 15–18 years from public and private high schools in Rawalpindi and Islamabad were selected for the study. A cross-sectional study design was incorporated and the data were gathered through the Diet Quality Questionnaire (DQQ), Kuppuswamy Socioeconomic Scale and Body Satisfaction and Image Questionnaire.

**Results:**

The analysis showed that 42.7 of % students possess a moderate diet and 57.3% possessed a poor diet. Diet quality was significantly associated with class grade, age, gender, socioeconomic status, body image and academic performance (*p* < 0.001). Students with a lower socioeconomic status, girls, senior grade students, and younger students were more likely to have poor eating habits.

**Conclusion:**

The findings present an urgent need for specific nutritional education and interventions in schools, in particular, among high-risk and vulnerable groups. Enhancing teenage diets can impact their psychological conditions, intellectual growth, and overall school performance.

## 1 Introduction

Adolescence is a period of rapid physical and mental development. Maintaining a healthy and adopting a balanced diet needs to be prioritized. It is essential for overall health as it affects the cognitive function and academic performance (1). The eating habits developed during this period can have a profound impact on future health-related conditions, such as the development of chronic diseases, including obesity, diabetes, and cardiovascular diseases (2). Additionally, teenagers go through various emotional and mental transformations, which can increase their vulnerability to unhealthy food choices. Their eating habits may be further complicated by peer influence, media influence, and the desire to act independently when it comes to food choice (3). Therefore, the factors that influence diet quality in adolescence are critical to understand, prevent, and manage, the emergence of diet-related issues in adulthood (4). Moreover, the period of adolescence is the onset of greater freedom in terms of food choices, because adolescents begin to make decisions beyond their family influence. Advertisements, social media, and peer influence tend to promote poor diets, especially those that are high in sugars, fats, and sodium (5).

The socioeconomic status has a great influence on the dietary habits of adolescents. Those living below the poverty line exhibit a higher risk of limited access to nutritious foods, resulting in a low-quality diet. Such a difference not only affects their physical wellbeing but may also negatively affect their cognitive growth and academic achievements (6). Young people with low-income families have limited purchasing power to buy fresh produce, low-calorie proteins, and other healthy alternatives, and instead prefer using cheaper, highly processed ones that contain high amounts of calories and lower nutritional value (7). Moreover, the economic pressure of these families can also act as a hindrance toward finding educational materials about nutrition, which only continues to promote poor eating habits (8). This is faced by the increased price of fresh and healthy foods over the processed, energy giving foods, and thus it is harder to ensure that low-income level families comply with the prescribed dietary requirements.

Another factor that has an impact on dietary behavior among adolescents is perceived body image. Individuals with poor body image perceptions are also more likely to engage in unhealthy eating habits, including restrictive diets or skipping meals, which further worsen their nutritional body conditions and academic performance (9). Media illustrations of the best type of body, which is unrealistic and not attainable by a lot of adolescents, tend to worsen body image dissatisfaction (10). Low body image may also result in emotional distress on the part of the adolescents who may decide to cope with this by either eating more or eating less. On the other hand, teenagers who have a more favorable body image will tend to consider food as a source of nutrition and not a way to change their appearance (9). Body positivity and self-acceptance promotion, as well as nutritional education, could be essential to enhancing food preferences and a healthy life in teenagers (11). Besides the psychological factor, the peer pressures exerted on adolescents to fit into a certain body shape might result in dieting habits and the adoption of weight control practices, including fasting or the use of diet pills. These practices not only affect the regular eating habits but may also cause severe long-term health effects, such as eating disorders, nutritional deficiency, and metabolic imbalances (12).

Adolescence is a developmental period in which the individual faces increased stress, peer pressure and social pressure. All these can predispose the individual to develop unhealthy eating patterns. Across 61 different countries, 17.9% of students were affected psychologically. The percentage was higher among girls compared to boys (20.8 vs. 14.9) (13). The lower consumption of vegetables and fruits, regular use of soft drinks and fast food were significantly related to stress, which shows the direct influence of the emotional strain on the food preferences. Similarly, the national statistics of Pakistan indicated that 38.8 percent of college students who suffered anxiety and 70 percent of those who suffered depression were associated with unhealthy eating habits that include a high level of saturated fats and refined carbohydrates (14). Such results highlight the impact of psychological distress and low-quality food on worsening mental health and physical conditions.

Another critical reason why adolescents are making poor dietary decisions is the prevalent advertisement of unhealthy foodstuffs across traditional and social media. Studies have shown that among children and teenagers, there is a constant exposure to commercials and advertisements about fast foods, highly processed foods and sugar-sweetened beverages, which subsequently greatly influence their 2eating habits and tastes. The most promoted ones were fast food restaurants and sugary drinks, and 83% of food and beverage ads by social media popular people in Canada were classified as less healthy (15). In the same manner, a narrative review that summarized 25 years of studies found that more than 90 percent corroborated the positive relationship between exposure to unhealthy food marketing and increased consumption of marketed products (16). All these, along with the high impact of multi-platform and influencer-led marketing, make unhealthy foods an ordinary good and lead to diet-related non-communicable diseases, indicating that it is high time to implement regulatory measures to safeguard the youth who are at risk. The rates of adolescents aged 13–15 years who eat fruits and vegetables at least five times per day are very low, as shown by data from the Global School-based Student Health Survey (GSHS): 12.6% in Libya and up to 38.1% in Djibouti. On the other hand, the percentage of adolescents who consume carbonated soft drinks daily was high, and it was reported as between 30.8 and 66.6 percent in the age group of 13–14 years and 31.5 to 56.9 years, respectively (17). These statistics show that there is a significant inclination toward processed and sugary types of food rather than whole foods. Consumption of carbonated soft drinks is a matter of great concern mainly because such drinks are associated with a higher risk of obesity, diabetes, and tooth decay. There can be many reasons that might lead to such a low intake of fruits and vegetables, such as the poor availability of fresh products, the absence of education about the value of such products, and the tendency to use more energy-dense and processed foods influenced by culture (18). Besides these external factors, there exists another issue of concern that is connected to the marketing efforts of food and beverage companies that tend to target youth in advertising their sugary snacks and beverages (19). The popularity of such advertisements in the numerous media networks, such as social media, television, and billboards, also contributes to the poor choice of food. Addressing the problem of low consumption of healthy foods and promoting healthy eating among adolescents it is possible with the help of effective policy interventions, including stricter regulations on food advertising and promotion of healthier options (20).

Studies throughout the EMR also indicate the poor dietary practices of the adolescents. Research has revealed that there is a range of 11 to 33.5 percent daily consumption of fruits among adolescents in most countries, with the exception of Iraq and Palestine which were reported to have higher rates. The consumption of vegetable diets is also different, with Bahrain, Jordan, Kuwait, Morocco, Qatar and Saudi Arabia recording 20 to 43 percent per day and Egypt (78.2), Iraq (46–62), Palestine (73) and Sudan (70) recording higher percentages. The difference in the consumption of fruits and vegetables between such countries highlights that the culturally-focused interventions should consider the local dietary preferences and socioeconomic determinants (21). Moreover, the dietary habits and cultural standards have an important influence on the adolescent eating habits in a region. In other regions, traditional food might not contain adequate amounts of fruits and vegetables, and others might be exposed to modernized food that is highly dependent on fast food and processed food (22). Consequently, health leadership bodies in the regions need to come up with nutrition education programs that are within the local setting to take cover of traditional and modern day eating issues so as to enhance the nutritional status of adolescents in different cultures.

As compared to the national level of 13.5, the Minimum Dietary Diversity (MDD) is also low and is reported at 11.7 in Pakistan. The percentage of underweight adolescents is equal to the country (11.4 vs. 12.1), but the level of anemia is lower (56.6 vs. the national level of 43.8). These percentages show the continuous problem of poor nutrition in rural areas of Pakistan and the urgent need for specific interventions to enhance nutrition diversity and intake levels of micronutrients among teenagers (23). In Islamabad, 68.6% of the adolescents like the food available in the canteens during the break time, and 49% reported dissatisfaction with the food options present in canteens (24). The emphasis on food choice in schools suggests the importance of the change of school meal programs, which might be a key factor in the nutritional conditions of adolescents. It can be effective to change the dietary patterns of adolescents by improving the quality and the range of food sold in school canteens and raising the level of nutrition awareness (25). Also, the school is a setting where healthy eating practices are critical to be promoted. Since adolescents spend a large percentage of their day at school, they should be able to have healthy food choices. Healthy eating could be supported by policy interventions, which require the delivery of healthy foods and snacks, among other elements, and the implementation of nutrition education programs (26). When adequately put into practice, school-based nutrition interventions can reach many adolescents and promote the adoption of healthy eating habits throughout their lives.

The study investigates the relationship between the dietary quality, socioeconomic status, perceived body image, and academic success among high school adolescents in Rawalpindi and Islamabad. Given the fact that the impact of the diet quality on health outcomes is widely-reported, there is no coherent research on the relationship of diet patterns with socioeconomic factors and their overall influence on academic achievement and self-esteem. Researching the impact of socioeconomic status on dietary behavior and its consequential effects on academic performance and body image, in turn, will offer valuable information to policymakers and teachers. It assists in creating particular interventions that address the nutritional deficiency in the diet, improving performance in school, and developing a positive body image. Consequently, it will affect the creation of more appropriate policies and the development of education on public health which can be adopted in this respect and applied to meet the needs of adolescents.

## 2 Materials and methods

### 2.1 Study settings

The research was carried out in both the public and private high schools of Rawalpindi and Islamabad, Pakistan. These cities have been chosen because they represent different socioeconomic and educational statuses. It provides a good sample for assessing the diet quality of adolescents.

### 2.2 Study population

The targeted population was adolescents aged between 15 and 18 years. This represents late adolescence, the stage where socioeconomic status and body image are important in determining nutritional practices.

### 2.3 Study period

The study period was 6 months, starting from January 2025 to June 2025. The planning stage involved ethical approvals in January 2025. After that school permissions were obtained from school authorities, in the period between February and April 2025. Then data entry, data cleaning, and data analysis were carried out in May and June 2025.

### 2.4 Study design

The study design was a cross-sectional in order to measure the quality of the diet, the socioeconomic status, and body image satisfaction of the respondents at a single time. The data collection instrument was a structured questionnaire. This design permitted determining the correlations between variables without controlling the study environment.

### 2.5 Sample size calculation

The size of the sample was calculated with the help of the World Health Organization (WHO) sample size calculator and it was assumed that the confidence interval is 95 percent (Z = 1.96), the margin of error is 5 percent, and the prevalence is 0.788 according to past studies (27). The size of the sample needed was 255. The sample size of 281 students was used to take into consideration 10% of non-response or attrition that was expected in the study to make the statistical analysis robust.


$$n^{0}=\frac{z^{2}\mathrm{pq}}{e^{2}}$$

Where:

- n^₀^ = required sample size
- z = standard normal deviate corresponding to the desired confidence level (1.96 at 95% CI)
- p = estimated prevalence (0.788 based on (27))
- q = (1 − p) = 0.212
- e = margin of error (0.05)

Substituting values:

n^0^ = (1.96)^2^·(0.788) (0.212)/(0.05)^2^.

n^0^ = 3.8416·0.1669/0.0025.

n^0^ = 0.641/0.0025 = 256.4.

Thus, the minimum required sample size was ≈255 participants.

To account for a potential 10% non-response or attrition rate:

n^0^ = 256.4 + 25.6 = 282.

The final sample size was set at 281 adolescents, which adequately met the requirement for statistical robustness.

### 2.6 Sampling technique

**Figure 1:**
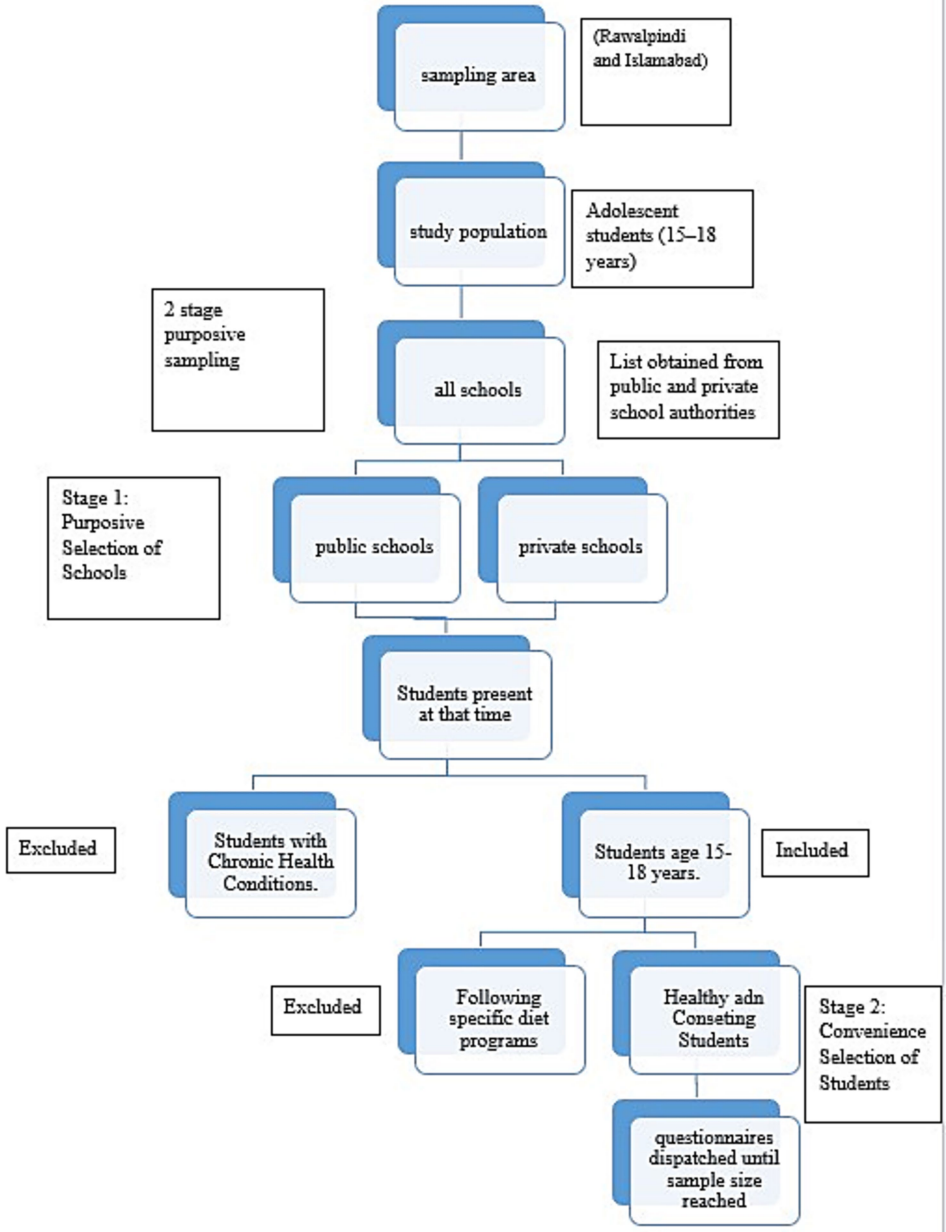
School selection process. The flowchart illustrates the sampling procedure, beginning with the selection of the study area (Rawalpindi and Islamabad) and the identification of the study population (adolescents aged 15–18 years). From this population, public and private schools were purposively selected, followed by the convenience selection of students who met the inclusion criteria. The final step shows that questionnaires were administered until the required sample size of 281 participants was achieved.

As shown in Figure 1, this study employed a two-stage purposive sampling technique. In the first stage, schools were purposively selected based on accessibility and willingness to participate. As shown in Table 1, a total of 10 schools were included: five public and five private, distributed across Rawalpindi and Islamabad to capture both urban and suburban representation. In the second stage, a convenience sampling approach was applied within each selected school (28). Students aged 15–18 years who were present on data collection days, gave informed consent (and parental consent), and met the study criteria were included.

**Table 1:** Participants’ distribution among selected schools.

School code	City	Type	Boys (N)	Girls (N)	Total (N)
A	Rawalpindi	Public	12	18	30
B	Rawalpindi	Public	10	15	25
C	Rawalpindi	Private	14	21	35
D	Rawalpindi	Private	11	14	25
E	Islamabad	Public	13	17	30
F	Islamabad	Public	16	19	35
G	Islamabad	Private	15	20	35
H	Islamabad	Private	12	18	30
I	Rawalpindi	Public	14	16	30
J	Islamabad	Private	11	25	36
Total			128	183	281

### 2.7 Inclusion criteria

- Adolescents aged 15–18 years enrolled in public or private schools of Rawalpindi and Islamabad.
- Students able to read and understand Urdu and/or English.
- Participants present during the data collection period (February to April 2025) and who provided informed consent.
- Adolescents not enrolled in any structured weight loss or diet program at the time of the study.
- Students who had not received a formal nutritional education program within the past year, to avoid bias in dietary knowledge and behavior.

### 2.8 Exclusion criteria

- Adolescents with chronic health conditions such as diabetes, cardiovascular disease, celiac disease, or severe allergies that could significantly affect dietary patterns.
- Students who had relocated to Rawalpindi or Islamabad within the last 6 months, as environmental and cultural exposure may differ from long-term residents.
- Participants who declined to provide consent or were absent during data collection days.
- Adolescents currently under a medically prescribed diet or specialized nutritional intervention.

### 2.9 Data collection procedures

Prior to data collection, ethical approval was obtained from the Institutional Review Board (IRB), ensuring adherence to ethical standards for human subject research. Formal permission was also obtained from school authorities. The schools were contacted via phone and email, shared study information, and conducted meetings with principals to clarify study goals. Once approval was granted, students and their guardians (where applicable) were informed about the study’s purpose and provided written consent. Data collection was conducted onsite. Students first filled out a demographic information sheet, followed by the study instruments. Participants were provided with clear verbal and written instructions, and all questionnaires were completed and collected during the same session to ensure completeness and data integrity.

### 2.10 Data collection tools

#### 2.10.1 Diet quality questionnaire

The Diet Quality Questionnaire (DQQ) was used to assess dietary patterns of adolescents. It is a valid tool developed by the Global Diet Quality Project to standardize the assessment of diet quality across populations (29). It consists of 29 yes/no questions regarding food and drink consumption during the previous 24 h, covering core food groups (grains, pulses, fruits, vegetables, and animal-based foods), discretionary foods (fried foods, sweets, and snacks), and beverages (milk, tea, juices, and carbonated drinks). The tool provides dietary diversity indicators and allows estimation of diet quality by categorizing participants into poor, moderate, or good dietary intake based on the number of food groups consumed (30). Responses were coded as “1” for consumption and “0” for non-consumption, and scores were summed to reflect overall dietary diversity.

#### 2.10.2 Kuppuswamy’s socioeconomic status scale

Socioeconomic status was assessed using the modified Kuppuswamy’s Socioeconomic Scale, which classifies households based on three domains:

1) Education of the head of family (illiterate to professional degree, score 1–7),
2) Occupation of the head of family (unemployed to legislators/senior officials, score 1–10), and
3) Monthly family income updated to 2023 Pakistani Rupees (score 1–12).

The total score ranges from 3 to 29 and categorizes families into five socioeconomic classes: Upper (26–29), Upper Middle (16–25), Lower Middle (11–15), Upper Lower (5–10), and Lower (<5). This tool is validated and widely used in South Asian populations for socioeconomic stratification (31).

#### 2.10.3 Body self-image questionnaire

Body image perception and satisfaction were assessed using the Body Self-Image Questionnaire (BSIQ), which includes 27 items rated on a Likert scale. The tool was developed by Rowe and his colleagues in 1999 (32). It was shortened by Rowe in 2015 for further development.

The tool comprises nine subscales:

- Overall Appearance Evaluation (OAE)—satisfaction with body appearance,
- Health Fitness Influence (HFI)—effect of health/fitness on self-image,
- Investment in Ideals (II)—importance of achieving ideal body standards,
- Health-Fitness Evaluation (HFE)—perceptions of health and fitness levels,
- Attention to Grooming (AG)—emphasis on grooming and appearance,
- Height Dissatisfaction (HD)—concerns about height,
- Fatness Evaluation (FE)—perception of fatness,
- Negative Affect (NA)—negative emotions about body image, and
- Social Dependence (SD)—influence of social comparisons on body image.

Each item is scored according to its response category, and subscale scores are summed to reflect the psychological and perceptual domains of body self-image. Higher scores on negative domains (e.g., FE, NA, HD, and SD) indicate greater dissatisfaction, whereas higher scores on positive domains (e.g., OAE, HFE, and AG) reflect a more favorable body image (33).

### 2.11 Statistical analysis

All collected data were coded and entered into SPSS version 27 for analysis. Descriptive statistics were generated to summarize demographic data, socioeconomic status, dietary quality indicators, and body image perceptions. To assess associations between variables such as dietary quality, socioeconomic status, body image satisfaction, and academic performance, chi-square tests were conducted. Prior to analysis, the dataset was checked for completeness, missing values, and coding consistency. Categorical responses (e.g., DQQ and BSIQ items) were numerically coded, and ordinal responses from Likert scales were appropriately handled. A significance level of *p* < 0.05 was used for inferential testing.

## 3 Results

The results include demographic characteristics such as gender, age, class level, academic performance, and socioeconomic status. These variables were assessed to explore their potential associations with diet quality.

As shown in Table 2, out of the total 281 participants, 117 (41.6%) were male and 164 (58.4%) were female. The age distribution indicated that the majority, 197 (70.1%), were in the 17–18-year age group. Regarding academic level, 131 (46.6%) participants were enrolled in 2nd year or A-Level programs. In terms of academic performance, 92 (32.7%) students scored an A grade (≥85%), while 114 (40.6%) achieved a B grade (≥75%). The majority of participants, 151 (53.7%), belonged to the Upper Middle (II) socioeconomic class, whereas 9 (3.2%) and 7 (2.5%) were classified under the Lower Middle (III) and Upper Lower (IV) categories, respectively.

**Table 2:** Descriptive statistics of the participants.

Variable	Category	Frequency (n)	Percentage (%)
Gender	Female	164	58.4
	Male	117	41.6
Age group	15–16 years	84	29.9
	17–18 years	197	70.1
Class	9th	24	8.5
	10th / O Levels	44	15.7
	1st Year	82	29.2
	2nd Year / A Levels	131	46.6
Academic grades	A (Above 85%)	92	32.7
	B (Above 75%)	114	40.6
	C (Above 65%)	52	18.5
	D (Above 55%)	23	8.2
Socioeconomic status	Upper (I)	111	39.5
	Upper Middle (II)	151	53.7
	Lower Middle (III)	9	3.2
	Upper Lower (IV)	7	2.5
	Lower (V)	3	1.1

Table 3 provides a detailed report on the frequency distribution of dietary quality indicators. The Non-Communicable Disease (NCD)-Risk score was 1.8, indicating that, on average, each adolescent consumed nearly two unhealthy food items such as sugary drinks, sweet snacks, or fried foods. Conversely, the NCD–Protect score stood at 3.1, which suggests the presence of three positive dietary behaviors, including the intake of legumes, fruits, and animal-source foods. One of the essential DQQ indicators, known as *All-5*, assesses whether individuals consumed at least one item from all five core food groups within the last 24 h. In this sample, 101 (36.0%) adolescents met this criterion. Furthermore, 121 (43.1%) consumed at least one vegetable, and 104 (37.0%) consumed at least one fruit. A high proportion, 261 (92.9%), reported intake of at least one item from the pulses, nuts, or seeds group, while 200 (71.2%) consumed an animal-source food. In contrast, only 101 (36.0%) reported intake of starchy staples. Alarmingly, 160 (57.0%) adolescents reported zero vegetable or fruit consumption on the assessed day. Additionally, 138 (49.1%) consumed sweet foods, 84 (29.9%) consumed salty or fried snacks, 84 (29.9%) consumed deep-fried items, and 76 (27.0%) reported whole grain consumption.

**Table 3:** Frequency distribution of dietary quality indicators overall.

Indicator	Scores/Percentages
GDR (Global Dietary Recommendations) Score	10.4
NCD-Risk Score (Negative dietary behaviors)	1.8
NCD-Protect Score (Positive dietary behaviors)	3.1
All-5 (Minimum consumption of all five core food groups)	36%
At least one vegetable	43%
At least one fruit	37%
At least one pulse, nut, or seed	93%
At least one animal-source food	71%
At least one starchy staple	36%
MDD-W (Minimum Dietary Diversity for Women)	–
Dietary Diversity Score (DDS)	4
Zero vegetable or fruit consumption (−)	57%
At least one vegetable or fruit	43%
Pulse consumption	69%
Nuts or seeds consumption	54%
Whole grain consumption	27%
Processed meat consumption (−)	19%
Salty or fried snack consumption (−)	30%
Deep fried food (−)	30%
Sweet foods consumption (−)	49%
Soft drinks (sodas, energy drinks, sports drinks) (−)	11%

**Figure 2:**
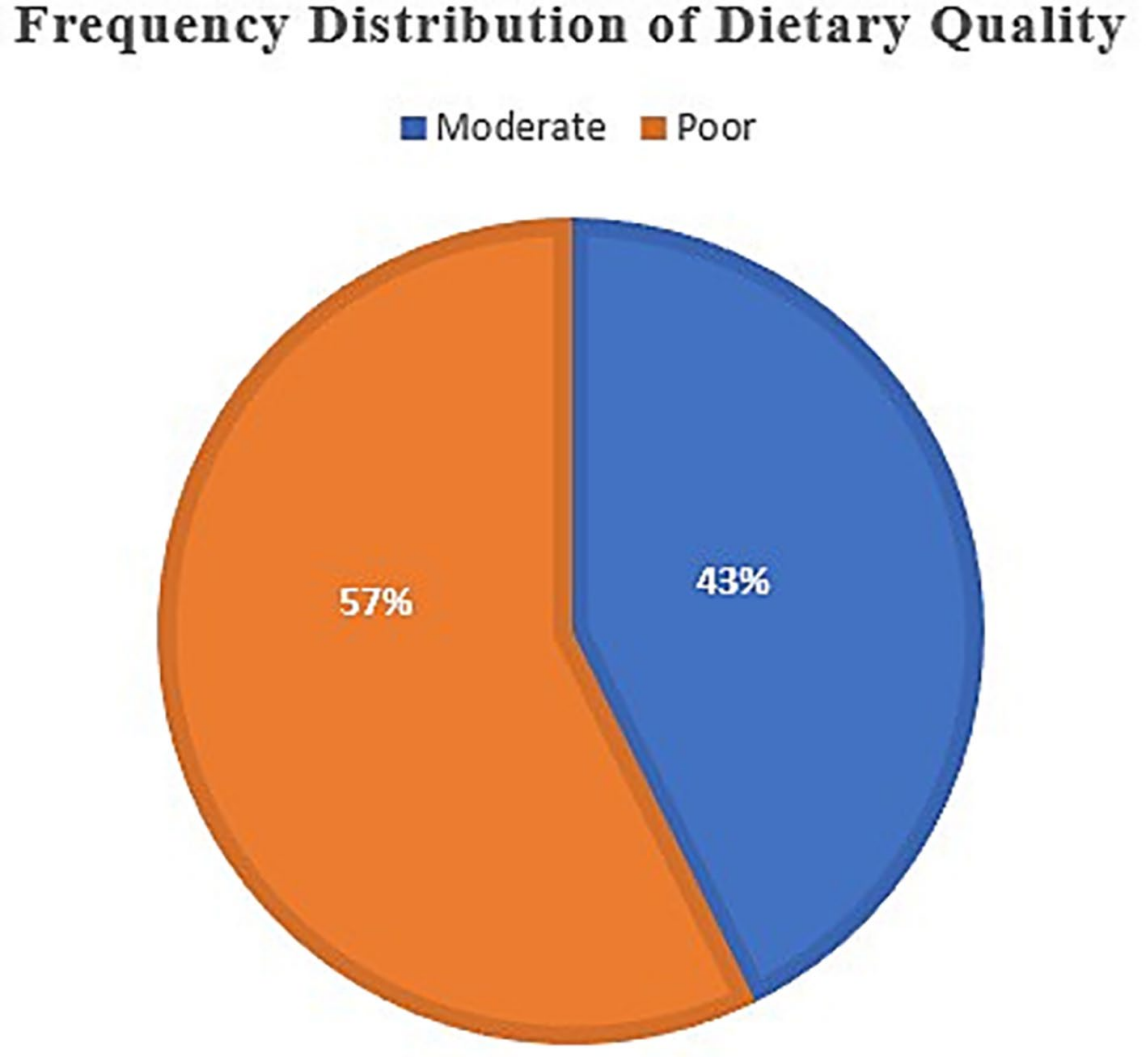
Frequency distribution of dietary quality among adolescents. Among the participants, 57% reported poor diet quality, while 43% reported moderate diet quality. These findings indicate that the majority of adolescents follow suboptimal dietary patterns, reflecting potential risks for academic and health outcomes.

Figure 2 reports the distribution of diet quality among a sample of 281 school-going adolescents from Rawalpindi and Islamabad. According to the results, 42.7% of the adolescents (*n* = 120) fell into the “moderate” diet quality category, 57.3% (*n* = 161) were identified as having a “poor” diet quality.

**Figure 3:**
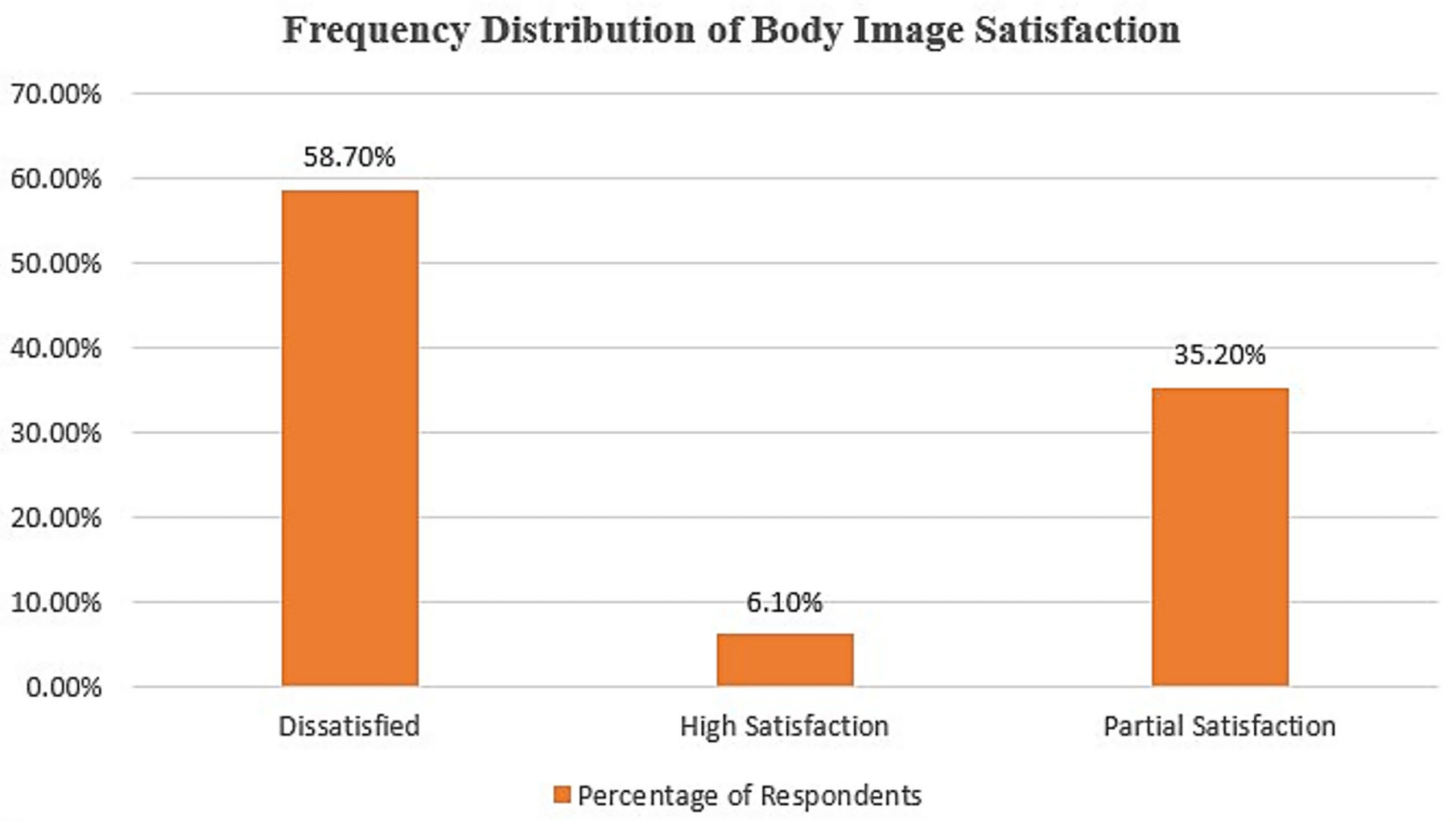
Frequency distribution of body image satisfaction among adolescents. The majority of participants (58.7%) reported dissatisfaction with their body image, while 35.2% expressed partial satisfaction. Only a small proportion (6.1%) indicated high satisfaction, highlighting overall concerns with body image in this population.

Figure 3 displays the frequency distribution of body image satisfaction. Among 281 adolescents surveyed in this study, 165 (58.7%) reported dissatisfaction with their body image, while 99 (35.2%) expressed partial satisfaction. Only 17 (6.1%) of the respondents, after merging the “High Satisfaction” and unclassified responses, expressed high satisfaction with their body image.

Table 4 summarizes the associations between various demographic factors and diet quality among 281 participants, categorized into moderate and poor diet quality groups. A significant relationship was found between age group and diet quality (χ^2^ = 13.45, *p* = 0.000). Participants aged 15–16 years (50 out of 84; 59.5%) had a higher proportion of poor diet quality compared to those aged 17–18 years (111 out of 197; 56.3%), indicating that younger adolescents were more likely to exhibit poorer dietary habits. Gender was also significantly associated with diet quality (χ^2^ = 13.93, *p* = 0.0003). Females were more represented in the poor diet quality category, with 107 out of 164 (65.2%) experiencing poor diet quality compared to 54 out of 117 (46.2%) males. Class grade was another demographic variable significantly associated with diet quality (χ^2^ = 13.55, *p* = 0.000). Students in the 2nd Year/A Levels class (77 out of 131; 58.8%) had the highest number of participants with poor diet quality, whereas 9th grade students (17 out of 42; 40.5%) showed relatively fewer cases of poor diet quality. This suggests that as students’ progress to higher academic levels, their risk of developing poorer dietary habits increases. Socioeconomic status also showed a strong and significant association with diet quality (χ^2^ = 30.99, *p* = 0.0002). Participants from lower socioeconomic groups, particularly those in the Lower (V) category (26 out of 34; 76.5%) and Upper Lower (IV) category (58 out of 85; 68.2%), had a higher prevalence of poor diet quality compared to those in higher socioeconomic brackets.

**Table 4:** Association between demographic variables and diet.

Demographic variable	Category	Moderate diet quality	Poor diet quality	Total	Chi-square (df)	*p*-value
Age group (years)	15–16	34	50	84	χ^2^ = 13.45 (1)	0.000*
	17–18	86	111	197		
	Total	120	161	281		
Gender	Female	57	107	164	χ^2^ = 13.93 (1)	0.0003*
	Male	63	54	117		
	Total	120	161	281		
Class grade	9^th^	13	11	24	χ^2^ = 13.55 (3)	0.000*
	10th / O Levels	21	23	44		
	1st Year	32	50	82		
	2nd Year / A Levels	54	77	131		
	Total	120	161	281		
Socioeconomic status	Lower (V)	24	60	84	χ^2^ = 30.99 (4)	0.0002*
	Lower Middle (III)	7	11	18		
	Upper (I)	8	13	21		
	Upper Lower (IV)	72	83	155		
	Upper Middle (II)	9	14	23		
	Total	120	161	281		

^*^Means statistically significant values.

Table 5 displays the association between diet quality and academic performance among the 281 participants. Of the total, 122 (43.4%) students reported moderate diet quality, while 159 (56.6%) reported poor diet quality. A chi-squared test of independence was conducted to examine whether an association exists between academic performance and diet quality. The results revealed a statistically significant relationship between the two variables (χ^2^ = 56.06, df = 3, *p* < 0.001).

**Table 5:** Association between diet quality and academic performance.

Academic performance	Moderate diet quality	Poor diet quality	Total	Chi-square (df)	*p*-value
A (Above 85%)	44	48	92	χ^2^ = 56.06 (3)	0.000*
B (Above 75%)	50	63	113		
C (Above 65%)	20	34	54		
D (Above 55%)	8	14	22		
Total	122	159	281		

^*^Means statistically significant values.

Table 6 illustrates the relationship between diet quality and satisfaction levels among 281 participants. A chi-squared test of independence was performed to explore the association between diet quality and satisfaction levels among 281 students. The Chi-Square statistic was 22.46, with 2 degrees of freedom, and the *p*-value was 0.0007.

**Table 6:** Association between body self-image and diet quality.

Body self-image	Moderate diet quality	Poor diet quality	Total	Chi-square (df)	*p*-value
Dissatisfied	25	38	63	χ^2^ = 22.46 (2)	0.0007*
High satisfaction	62	91	153		
Partial satisfaction	33	32	65		
Total	120	161	281		

^*^Means statistically significant values.

## 4 Discussion

This study aimed to evaluate the association between diet quality and academic performance among adolescents aged 15–18 in Rawalpindi and Islamabad, Pakistan. The sample size of 281 participants was chosen using a convenience sampling method in both the public and private schools. The study investigated the relationship between diet quality and academic achievement, body image satisfaction, and socioeconomic status (SES). The Diet Quality Questionnaire (DQQ), the Body Satisfaction and Image Questionnaire (BSIQ) and the Kuppuswamy Socioeconomic Scale were used to gather self-reported measures. Following the data analysis based on chi-square tests of independence, the statistically significant association of diet quality with all three major variables was observed, which indicates that eating habits contribute to the multidimensional influences on the development of cognitive, emotional, and social outcomes during the adolescent stage.

In our research, the demographic variables showed significant trends. The adolescents who were younger (15–16 years old) reported lower-quality diets than older individuals, which is contrary to another study which discovered that older adolescents were more likely to eat conveniently (34). Such a difference can be attributed to regional and cultural differences in autonomy, peer pressure and parental interferences in food preferences. There were also gender differences and females reported a low quality of diet as compared to males. On the same note, another report observed that restrictive eating habits in adolescent girls can be promoted because of sociocultural pressures which could be the explanation of the differences found in this study (35). According to another evidence, results of the Health Behavior in School-aged Children (HBSC) survey in 45 European countries found that the prevalence of meal skipping and dieting is higher in girls than in boys (36). In addition, another study demonstrated that body image strongly influences the dietary choices in adolescent girls, reinforced by evidence of gender-specific concerns (9).

Academic grade was the other determinant, where higher grades (2^nd^ Year/A levels) were associated with greater likelihood of poor diet quality. This observation is consistent with the fact that higher grade levels are linked with increased academic workload and stress, which results in poor eating habits (37). In the same way, the socioeconomic inequality to a large extent influenced the quality of food, with the poor population exhibiting a greater percentage of unhealthy food habits that contributed to poor nutrition. This correlates with the evidence that continuously associates economic reasons and a lack of access to nutritious food with unhealthy diets (18, 38). Such results indicate the necessity of a systemic intervention with the basis of affordability, accessibility, and food literacy.

Equally, it has been pointed out that the socioeconomic disparities in adolescence is associated with reduced dietary diversity and increased intake of energy-dense, nutrient-dense foods (39). Moreover, it is demonstrated that healthier diets are systematically more costly, and affordability is one of the critical obstacles of low-income families (40). Lastly, there was a strong correlation between the quality of the diet and academic performance. Students who had higher grades were more likely to eat healthier, which is also consistent with previous research that nutrient-rich foods improve memory, attention, and cognitive flexibility (41, 42). Poor diet quality, in turn, was associated with lower academic performance which could be explained by a decrease in concentration, fatigue, and less energy. This observation is also reinforced by the fact that the healthier the diet of adolescents, the better they performed in standardized literacy tests (43). Similarly, dietary patterns that are rich in fruits, vegetables, and whole grains have been observed to positively correlate with academic achievement, and intake of processed foods has been observed to predict poor performance in school (44). These results support the advantages of educating students about healthy eating in schools.

The quality of the dietary intake revealed that over 50% of the adolescents had poor dietary habits, involving less intake of fruits, vegetables, and whole grains along with excessive intake of unhealthy food. This tendency is consistent with the previous research in South Asia and other countries that has reported about a nutrition transition with a rise in the use of processed food (34). Likewise, one of the studies emphasized that limited access to healthy foods during adolescence could impede educational achievement and future wellness (41). There is another study that supports these findings, whereby more intakes of fast food and sugary beverages were associated with lower intakes of micronutrients and higher chances of overweight or obesity (45). Moreover, one study demonstrated that low nutritional quality was among the primary risk factors of untimely mortality, and adolescents are particularly vulnerable to it since they develop lifetime eating patterns (46). These findings highlight the necessity of immediate school-based nutritional programs to promote eating healthier foods.

Body image satisfaction was greatly related to the quality of diet. The healthier the diet was among adolescents, the more they were likely to report more body satisfaction, and the poorer the dietary practices, the more dissatisfaction. This result does not contradict a study where researchers proved that bad eating habits tend to support the negative view of body image (47). On the contrary, the balanced diets have been associated with improved mood, enhanced energy and enhanced self-perception. Likewise, one study in Spain discovered that emotional distress and problematic social media mediation mediated the association between unhealthy eating habits and increased body dissatisfaction, especially in females (48). Furthermore, another study mentioned that poor eating habits are also caused by stress, which, besides disrupting physical health, influences the perception of negative body images (49). Those findings demonstrate the psychosocial implications of eating habits and the need to incorporate body positivity into food-related educational practices.

In addition, it has been established that body dissatisfaction is associated with mental distress, particularly tension and anxiety, and problematic social media use is the mediator of this relationship (Monton, Diego, and Estevez, 2024). A meta-analysis states that stress can adversely affect eating behavior by raising consumption of harmful foods and reducing consumption of good foods (49). The implication of these results is that body dissatisfaction not only undermines mental health, but it can also indirectly result in the poor quality of the diet by triggering eating behaviors that are associated with stress. Moreover, the tastes of teens toward food are largely shaped by their exposure to social media, and celebrities and other influencers often push high-energy and nutrient-dense food, which strengthens unhealthy eating patterns (50). Strengths of the study include addressing a gap in the literature by investigating the nature of the relationship between the quality of diet and the outcome of academic performance among teenagers in South Asian countries. It also provides useful insights to a region and a very critical problem at a global level. The sample (n = 281) was sufficiently large to render the results strong, and the method of dietary assessment was followed by the utilization of the validated tools, such as the DQQ. In addition, the research study looks into the demographic and socioeconomic inequities in the quality of the diet that can be employed to set up school and community-based interventions. There are also few limitations. The causal interpretation was not that great, as the study design was cross-sectional. One of the limitations is the recall or reporting bias that could have been caused by the use of self-reported questionnaires. The research was limited to Rawalpindi and Islamabad, and thus the generalization to other areas is not possible. Moreover, other determinants like exercise, peer pressure, and mental status were not evaluated, which can also define dietary habits and academic performance.

## 5 Conclusion

This paper analyzed the relationship between the quality of diets, academic achievement, body self-image and socioeconomic status of adolescents in Rawalpindi and Islamabad. The results show that there are very strong correlations between these factors, indicating that the quality of the diet is a very important factor in academic performance and emotional health. The overall effect of low quality of diet on poor performance in schools helps to support the hypothesis that nutritional habits can have a direct or indirect impact on cognitive capabilities like concentration, memory retention, and general classroom performance. Moreover, the body self-image was also largely connected to the quality of diet, and students who had a moderate diet had a higher level of satisfaction with their body image. Nutrition education programs should be incorporated in the school’s curriculum so as to sensitize them on the advantages of eating healthy food. Such programs can be especially productive when it comes to reaching students who belong to the lower SES group and might lack access to healthy diets and food options at home. The gap can be closed by ensuring that such meals are accessible and affordable to students whose families have low incomes. Involvement of parents in nutritional education can assist in supporting the idea of healthy eating at home. This may involve collaboration with local communities, non-governmental organizations and government schemes that pay attention to food aid. There is a need for further research in the areas of long-term quality of diet in academic and mental health outcomes. Longitudinal studies that track the impact of dietary changes over time could provide deeper insights into how nutrition influences adolescents’ cognitive and emotional development.

## Data Availability

The raw data supporting the conclusions of this article will be made available by the authors, without undue reservation.
